# [^18^F]fallypride characterization of striatal and extrastriatal D_2/3_ receptors in Parkinson's disease

**DOI:** 10.1016/j.nicl.2018.02.010

**Published:** 2018-02-10

**Authors:** Adam J. Stark, Christopher T. Smith, Kalen J. Petersen, Paula Trujillo, Nelleke C. van Wouwe, Manus J. Donahue, Robert M. Kessler, Ariel Y. Deutch, David H. Zald, Daniel O. Claassen

**Affiliations:** a Neurology, Vanderbilt University Medical Center, Nashville, TN, United States; b Psychology, Vanderbilt University, Nashville, TN, United States; c Radiology and Radiological Sciences, Vanderbilt University Medical Center, Nashville, TN, United States; d Psychiatry and Behavioral Sciences, Vanderbilt University Medical Center, Nashville, TN, United States; e Radiology, University of Alabama at Birmingham, Birmingham, AL, United States; f Pharmacology, Vanderbilt University, Nashville, TN, United States

**Keywords:** BP_ND_, Binding potential (nondisplaceable), CES-D, Center for Epidemiologic Studies Depression Scale, LEDD, Levodopa Daily Dose, MDS-UPDRS, Movement Disorders Society-United Parkinson's disease Rating Scale, MoCA, Montreal Cognitive Assessment, HC, Healthy controls, PD, Parkinson's disease, PET, Positron emission tomography, ROI, Region of Interest, Parkinson's disease, Dopamine, Positron emission tomography (PET), Neurodegeneration, Fallypride

## Abstract

Parkinson's disease (PD) is characterized by widespread degeneration of monoaminergic (especially dopaminergic) networks, manifesting with a number of both motor and non-motor symptoms. Regional alterations to dopamine D_2/3_ receptors in PD patients are documented in striatal and some extrastriatal areas, and medications that target D_2/3_ receptors can improve motor and non-motor symptoms. However, data regarding the combined pattern of D_2/3_ receptor binding in both striatal and extrastriatal regions in PD are limited. We studied 35 PD patients off-medication and 31 age- and sex-matched healthy controls (HCs) using PET imaging with [^18^F]fallypride, a high affinity D_2/3_ receptor ligand, to measure striatal and extrastriatal D_2/3_ nondisplaceable binding potential (BP_ND_). PD patients completed PET imaging in the off medication state, and motor severity was concurrently assessed. Voxel-wise evaluation between groups revealed significant BP_ND_ reductions in PD patients in striatal and several extrastriatal regions, including the locus coeruleus and mesotemporal cortex. A region-of-interest (ROI) based approach quantified differences in dopamine D_2/3_ receptors, where reduced BP_ND_ was noted in the globus pallidus, caudate, amygdala, hippocampus, ventral midbrain, and thalamus of PD patients relative to HC subjects. Motor severity positively correlated with D_2/3_ receptor density in the putamen and globus pallidus. These findings support the hypothesis that abnormal D_2/3_ expression occurs in regions related to both the motor and non-motor symptoms of PD, including areas richly invested with noradrenergic neurons.

## Highlights

• [^18^F]fallypride allows concurrent evaluation of striatal and extrastriatal D_2/3_.
• PD patients express widespread D_2/3_ reductions relative to healthy controls.
• Reductions are apparent in brain regions dominated by dopamine and norepinephrine.
• Percent reductions are larger in extrastriatal areas relative to the basal ganglia.
• D_2/3_ in the putamen and globus pallidus correlates with PD motor symptom severity.

## 1.1 Introduction

D_2_ and D_3_ receptors are expressed in high abundance in the striatum and ventral midbrain, and in lower levels in certain limbic and cortical regions (Gurevich and Joyce, 1999). Dopamine agonists that preferentially target these D_2/3_ receptors improve motor symptoms in Parkinson's disease (PD) (Shannon et al., 1997), and have been suggested to also reduce certain non-motor symptoms, such as depression (Barone et al., 2006). Early in the course of PD, striatal D_2/3_ binding potential (BP_ND_) increases (Rinne et al., 1993; Rinne et al., 1995), potentially due to reduced receptor occupancy by endogenous dopamine, or post-synaptic sensitization induced increases in receptor expression (Knudsen et al., 2004). This upregulation of D_2/3_ receptors is more extensive in the putamen than the caudate nucleus, consistent with the earlier and more prominent dopaminergic denervation of the putamen in PD (Gibb and Lees, 1991). With longer disease duration, D_2/3_ expression diminishes throughout the striatum (Antonini et al., 1997). Less attention has been devoted to the effects of PD on extrastriatal D_2/3_ expression. Some studies have reported a decrease in D_2/3_ BP_ND_ later in the course of PD, including in the medial thalamus as well as anterior cingulate, inferior temporal, and ventromedial/dorsolateral prefrontal cortices (Kaasinen et al., 2003; Kaasinen et al., 2000; Ko et al., 2013).

Due to intrinsic differences in properties of SPECT and PET radioligands, few studies have been capable of concurrently examining striatal and extrastriatal D_2/3_ binding in PD patients. The fact that most radioligands are best suited towards quantitation of binding in striatal or extrastriatal sites, but not both at the same time, makes a comprehensive understanding of the relative magnitude of PD-induced D_2/3_ BP_ND_ changes across areas compared to healthy subjects difficult to capture. [^11^C]raclopride is effective at measuring striatal (and to an extent thalamic) D_2/3_ levels but cannot provide reliable estimates in most extrastriatal areas, while [^11^C]FLB-457 can assess extrastriatal regions but is not able to quantify binding in the striatum (as it does not reach equilibrium in a reasonable timeframe) (Farde et al., 1997; Hall et al., 1989). The D_3_ preferring ligand [^11^C]-(+)-PHNO has infrequently been used to estimate dopamine receptor binding in PD (Boileau et al., 2009; Payer et al., 2015), but cannot provide full characterization of limbic and cortical regions (Egerton et al., 2010).

[^18^F]fallypride ((*S*)-N-[(1 allyl-2-pyrrolidinyl)methyl]-5-(3[18F]fluoropropyl)-2,3-dimethoxybenzamide) is a high-affinity D_2/3_ radioligand that can provide accurate estimates of binding in both striatal and extrastriatal regions, allowing for concurrent estimation of dopamine D_2/3_ receptor levels (i.e. nondisplaceable binding potential, BP_ND_) throughout the brain (Kessler et al., 2000; Mukherjee et al., 2002). Furthermore, [^18^F]fallypride has been successfully applied in a PD cohort (Deutschlander et al., 2016). We examined regional [^18^F]fallypride binding in a large cohort of PD patients and age-matched healthy control (HC) subjects in order to simultaneously determine differences in striatal and extrastriatal D_2/3_ BP_ND_, with the goal of providing cortical and subcortical binding potentials that can be directly compared. As a secondary objective, we assessed if D_2/3_ BP_ND_ reflected motor severity in PD patients.

## 1.2 Methods

### 1.2.1 Participants

PD participants were recruited from the Movement Disorders Clinic at Vanderbilt University Medical Center. All met UK Brain Bank criteria for a diagnosis of PD and were prescribed levodopa and DA agonist medications (including pramipexole, ropinirole, and rotigotine) for relief of motor symptoms. Daily doses of dopamine replacement therapy were converted to levodopa equivalent dose (Tomlinson et al., 2010). Patients were excluded if they had an implanted deep brain stimulator, received antipsychotic treatments, suffered from comorbid neuropsychiatric, cerebrovascular, or cardiovascular disease, could not tolerate a brain MRI/PET study, or dopaminergic medication withdrawal. HC subjects (Dang et al., 2017; Dang et al., 2016) did not have a history of psychiatric illness, head trauma, substance abuse, diabetes, or medical condition that precluded MRI collection, nor could they use tobacco. No participants took psychostimulant or psychotropic medications (with an exception for occasional use of benzodiazepines as sleep medication) over the preceding 6 months, and did not consume excessive alcohol. Urine drug tests were administered to all participants to ensure the absence of amphetamine, barbiturates, cocaine, marijuana, or opiates.

A neurologic exam was performed on all participants, in order to exclude parkinsonism in HC subjects. PD patients completed part II of the Movement Disorders Society-United Parkinson's disease Rating Scale (MDS-UPDRS) (a self-reported assessment of the impact of PD on activities of daily living), and part III (an assessment of motor function in PD) in the Off-medication condition (Goetz et al., 2008; Weintraub et al., 2012). Dopamine medications were withheld for >40 h prior to PET imaging for DA agonists and >16 h for levodopa prior to PET imaging (the half-life of levodopa, ropinirole, and pramipexole are approximately 1.5, 6, and 8–12 h respectively (Bennett Jr and Piercey, 1999; Fabbrini et al., 1987; Tompson and Oliver-Willwong, 2009; Wright et al., 1997). Cognitive screening was performed using the Montreal Cognitive Assessment (MoCA) to rule out patients with frank dementia (Nasreddine et al., 2005), requiring a score of at least 22. In PD patients, depression was screened using the Center for Epidemiologic Studies Depression Scale Revised (CESD-R) (Radloff, 1977). The presence of medication-induced impulsive compulsive behaviors (ICBs) as a potential confounding factor was also assessed using a semi-structured interview with patient and partner.

Demographic and clinical features for PD patients (*n* = 35), as well as HC subjects (*n* = 31) are presented in Table 1. Both groups had a similar average age and sex distribution. The side of symptom severity (both onset and based on motor testing) was more prominent in the left hemi-body of PD patients, who expressed moderate PD progression with an average disease duration of 5.9 ± 3.9 years. Of this cohort, 17 had symptoms of Impulsive Compulsive Behaviors.

**Table 1:** Demographic and clinical evaluation from the two participant groups.

Variables	PD	HC	*p-*Value
N	35	31	
Sex (M/F)	24/11	21/10	0.94
Age (years)	61.8 ± 8.5	58.1 ± 11.3	0.17
Disease duration (years)	5.9 ± 3.9	n/a	–
CES-D	15.7 ± 8.7	n/a	–
Laterality score (− = left worse, + = right worse)	−2.45 ± 10.7	n/a	–
Left worse/right worse (individual)	22/13		
MDS-UPDRS			
Part II	21.8 ± 7.7	n/a	–
Part III (OFF)	30.0 ± 11.1	n/a	–
Dopamine replacement therapy			
Total LEDD (mg/day)	632.7 ± 418.7	n/a	–
Agonist single dose equivalent (mg/day)	103.9 ± 71.6	n/a	–

Data are shown as mean ± standard deviation.

MDS-UPDRS Part III conducted off medication (36 h for DAgonist and 16 for LDOPA).

PD: Parkinson's Disease.

AMNART: American version of the National Adult Reading Test.

CES-D: Center for Epidemiologic Studies Depression Scale.

MDS-UPDRS: Movement Disorders Society-United Parkinsons Disease Rating Scale.

BIS: Barratt Impulsivity Scale.

LEDD: Levodopa Daily Dose.

Written informed consent was obtained from all subjects, and the study was performed in accordance with the Institutional Review Board at Vanderbilt University, adhering to the ethical standards stipulated by the Declaration of Helsinki and its amendments.

### 1.2.2 Magnetic resonance imaging

MRI scans were completed prior to PET scans in order to provide high-resolution structural delineation. Both PD and HC subjects were scanned at 3.0T (Philips, Best, The Netherlands) using body coil transmission and 8-channel SENSE reception. All underwent a T_1_-weighted high-resolution anatomical scan (MPRAGE; spatial resolution = 1 × 1 × 1 mm^3^; TR/TE = 8.9/4.6 ms).

### 1.2.3 Fallypride PET data acquisition

[^18^F]fallypride was synthesized in the radiochemistry laboratory consistent with the synthesis and quality control procedures outlined by US Food and Drug Administration INDs 47,245 and 120,035. Data were collected on a GE Discovery STE PET/CT scanner. Serial scan acquisition began simultaneously with a 5.0 mCi slow bolus injection of [^18^F]fallypride (specific activity >3000 Ci/mmol). CT scans were collected prior to each of the three emissions scans for the purpose of attenuation correction. Together, the scans lasted approximately 3.5 h with two breaks of 15–20 min (beginning approximately 70 min and 135 min after the beginning of the scan, respectively) included for patient comfort. During breaks, patients remained at rest but were permitted to stretch. Data for PD and HC subjects were acquired using identical MRI and PET technical parameters, with the single exception of a slightly different PET acquisition time protocol for the second and third dynamic runs, although total scan duration was similar (Supplementary Table 1; see Dang et al. (2017); Dang et al. (2016)). In past PET studies, differences in acquisition time protocol were not found to be a significant confound (Buckholtz et al., 2010; Smith et al., 2016).

### 1.2.4 Fallypride PET data processing

Following attenuation correction and decay correction, serial PET scans were co-registered using Statistical Parametric Mapping software (SPM8, Wellcome Trust Centre for Neuroimaging, London, UK, http://www.fil.ion.ucl.ac.uk/spm/software/) to correct for motion across scanning periods with the last dynamic image of the first series serving as the reference image. The mean PET image produced by realignment was then co-registered to the subject's corresponding high-resolution T1 MRI image using FSL's FLIRT with 6 degrees of freedom (FSL v5.0.2.1, FMRIB, Oxford, UK).

D_2/3_ receptor levels were estimated using the simplified reference tissue model (SRTM) (Lammertsma et al., 1996), performed in PMOD software (PMOD Technologies, Zurich Switzerland) to measure [^18^F]fallypride binding potential(BP_ND_; the ratio of specifically bound [^18^F]fallypride to its nondisplaceable concentration as defined under equilibrium conditions). Voxel-wise estimates were generated using a published basis function fitting approach (Gunn et al., 1997) conducted in the PXMOD module of PMOD. The rate constants were specified as k_2a_ minimum = 0.006 min^−1^ and k_2a_ maximum = 0.6 min^−1^. Due to the very limited expression of D_2/3_ receptors in the cerebellum (Camps et al., 1989), it was selected as the reference region (Kessler et al., 2000; Kessler et al., 2005). Subject-space analyses were conducted by warping baseline BP_ND_ images to T1 space with the saved FSL FLIRT transform matrices. For voxel-wise analyses, subject-space BP_ND_ images were registered to Montreal Neurological Institute (MNI) space using FSL's FNIRT (FSL v5.0.2.1, FMRIB, Oxford, UK).

Voxel-wise analysis was first applied to assess putative subcortical and cortical differences between groups, with specific attention towards potential sub-regional distinctions in the broader mesiotemporal and prefrontal cortices. Next, subcortical regions of interest (ROIs), including the caudate (head), putamen (whole body), globus pallidus, ventral striatum, amygdala, hippocampus, ventral midbrain, thalamus, and cerebellum, were manually segmented on the T_1_-weighted MRI scans by a neuroradiologist (RMK) and neurologist (DOC) experienced in PET and MRI data analysis, and transferred to the co-registered PET studies through the FLIRT FSL transformation matrix. Manual segmentation methods followed established anatomical criteria, capturing the central portion of the selected region to gather the most representative sample. This method was selected in order to best avoid partial volume effects in densely arranged subcortical nuclei, and were applied so as to avoid the potential confound of inter-subject structural variability (Kessler et al., 2009). The caudate, putamen, and globus pallidus were manually drawn on axial slices approximately 2–12 mm above the ACPC line. The ventral striatum was segmented on coronal slices with the criteria of Mawlawi et al. (2001). The amygdala can be identified on axial slices 6–20 mm below the ACPC line, 12–28 mm lateral to the midline, and 2–12 mm behind the plane of the anterior commissure (Schaltenbrand and Wahren, 1998). To minimize partial voluming of the striatum, amygdala ROIs were defined 10–16 mm beneath the ACPC plane. The ventral midbrain was drawn on axial slices in 9–14 mm below the ACPC line, and the thalamus was segmented 2–12 mm above the ACPC line (Schaltenbrand and Wahren, 1998). The cerebellar ROI was drawn centrally within the structure to avoid partial voluming of ventral midbrain or cortical signal, and contained an approximately equal distribution of grey and white matter. Due to difficulty capturing the extent of the hippocampus using manual methods, it was defined using a previously published automatic subject space segmentation method (Asman et al., 2015). All ROIs were defined bilaterally. In order to account for potentially divergent structure size between groups, ROI volumes were collected and preserved for statistical analysis.

### 1.2.5 Hypothesis testing and statistical analysis

Group differences in demographic and clinical parameters, as well as ROI volume, were evaluated using Mann Whitney *U* tests. Sex differences were evaluated with a chi-square test. A voxel-wise analysis investigated PD-related group differences in D_2/3_ BP_ND_ across cortical and subcortical regions. Age and sex were included as covariates, due to previous evidence that these factors influence D_2/3_ receptor status (Mukherjee et al., 2002; Pohjalainen et al., 1998), and significance criteria consisted of an uncorrected *p* < 0.005. Multiple comparisons correction was accomplished by controlling cluster-level FDR at 0.05. This analysis was completed using SPM8. In a secondary, exploratory voxel-wise analysis utilizing identical statistical parameters, BP_ND_ maps for subjects expressing more severe PD symptoms on the right side of the body were flipped across the rostral-caudal axis of the axial plane. This step was completed in order to ensure that the more degenerated side of the dopaminergic system (contralateral to more severe motor deficits) was aligned for all subjects (Antonini et al., 1997).

To test the hypothesis that PD patients have different dopamine D_2/3_ receptor expression in specific striatal and subcortical extrastriatal areas, mean group regional [^18^F]fallypride BP_ND_ was analyzed via a general linear regression model (GLM), where within-ROI BP_ND_ was the dependent variable and PD status was the primary independent variable. Age and sex were included as covariates. ROI volume was also specified as a covariate, to ensure that any differences were not due to overall structural atrophy. False discovery rate (FDR) was controlled at 0.1 to correct for multiple comparisons, in concordance with the threshold recommended in the first description of the method (Benjamini and Hochberg, 1995). All analyses were performed using SPSS Statistics 24 (IBM, Armonk, NY, USA) and R (R Foundation for Statistical Computing, Vienna, 2016). In addition to the ROI analysis, we performed a post-hoc evaluation to quantify the magnitude of BP_ND_ differences in regions where voxel-wise distinctions were present, but were not captured by the hand-drawn ROI analysis. Mean group BP_ND_ values were extracted from standard space (MNI) in areas where voxel-wise clusters were manifested using the Automated Anatomical Labeling (AAL) atlas or manual segmentation methods. This served as a post-hoc method of evaluating the extent of regional differences identified by the voxel-wise analysis.

The relationship between regional [^18^F]fallypride BP_ND_ and PD severity was also examined using a partial Pearson's correlation covarying for age, sex, ROI volume, disease duration, and levodopa equivalent daily dose (LEDD). This process was repeated for both MDS-UPDRS Part II (a measure of activities of daily living) and MDS-UPDRS Part III-Off (a measure of motor symptom severity), and the results were controlled at FDR of 0.1 to correct for multiple comparisons. A secondary voxel-wise analysis was also completed for MDS-UPDRS Parts II and III using SPM8, specifying age, sex, disease duration, and LEDD as covariates, an uncorrected threshold of *p* < 0.005, and cluster-level FDR controlled at 0.05. This voxel-wise analysis was also repeated using images flipped across the y-axis of the axial plane to align the side of the brain contralateral to the side of greater PD motor symptom severity in all subjects.

## 1.3 Results

### 1.3.1 Voxel-wise fallypride binding potential differences

**Fig. 1:**
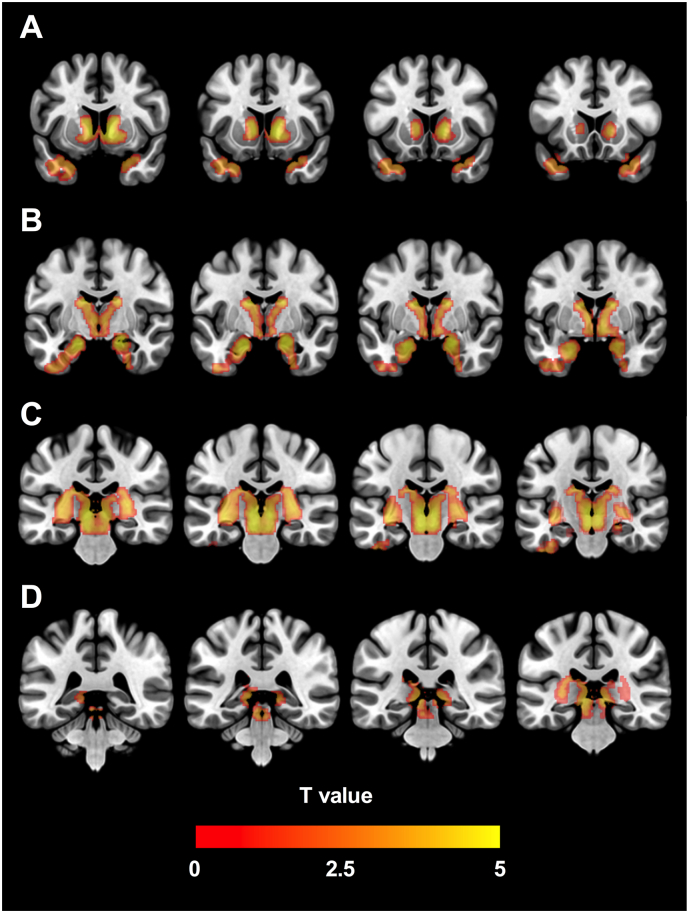
Voxel-wise [^18^F]fallypride binding potential analysis. Map of significant clusters where [^18^F]-fallypride BP_ND_ was reduced in PD, overlaid on coronal slices of an MNI template brain. All survived cluster-level FDR correction at *p* < 0.05, and localize to areas including (A) the striatum, globus pallidus, and temporal cortex, (B) the amygdala and hippocampus, (C) the ventral midbrain and thalamus, and (D) the locus coeruleus.

**Fig. 2:**
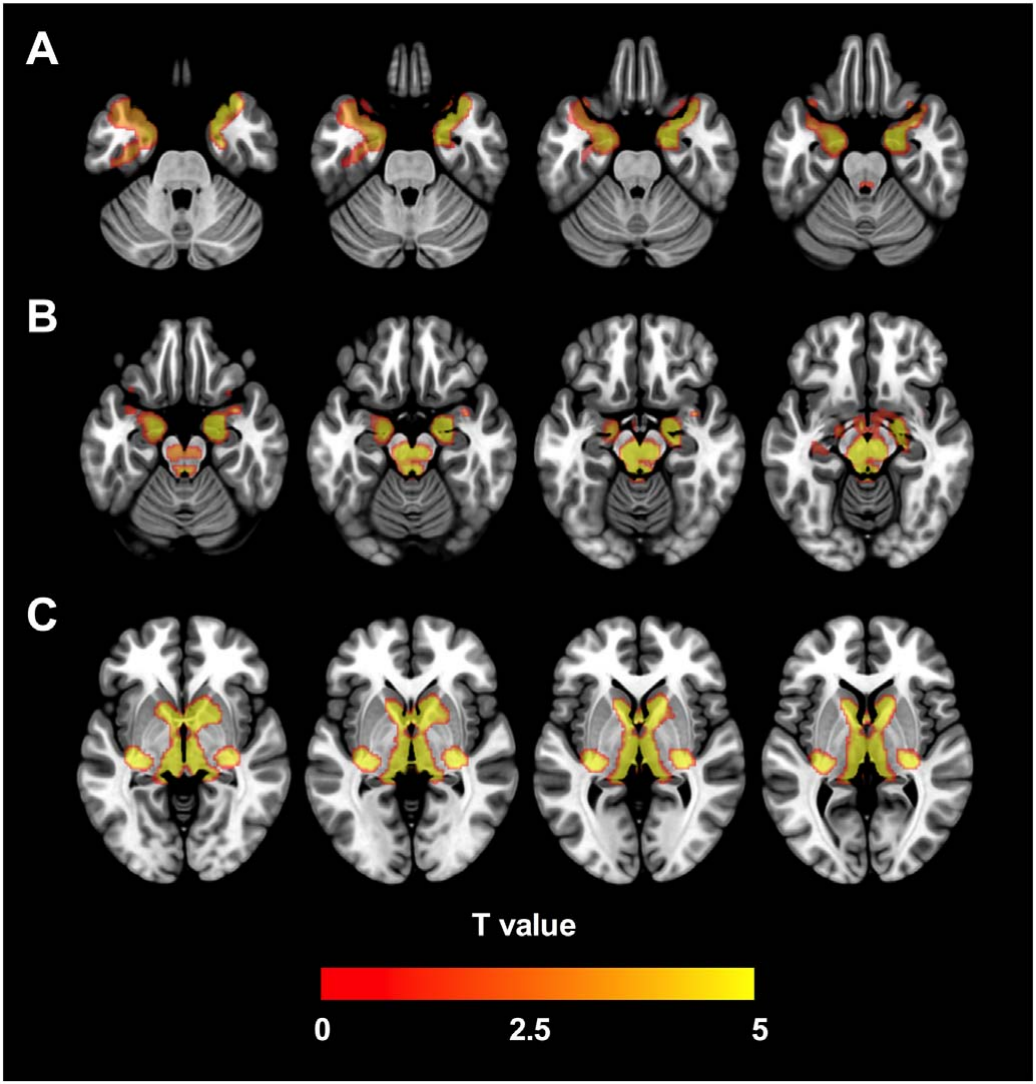
Voxel-wise [^18^F]fallypride binding potential analysis. Map of significant clusters where [^18^F]-fallypride BP_ND_ was reduced in PD, overlaid on axial slices of an MNI template brain. All survived cluster-level FDR correction at *p* < 0.05, and localize to areas including (A) the temporal cortex, (B) the amygdala, hippocampus, ventral midbrain, and locus coeruleus, and (C) the thalamus, striatum, and globus pallidus.

Using the voxel-wise method covarying for age and sex, significant reductions of [^18^F]fallypride BP_ND_ in PD patients localized to subcortical clusters in the amygdala and hippocampus, ventral midbrain, LC, caudate nucleus, and globus pallidus. Cortical differences localized to the anterior and medial temporal cortical regions (temporal pole, parahippocampal, and entorhinal cortices). In no region was D_2/3_ BP_ND_ increased in patients with PD compared to HC participants. Fig. 1, Fig. 2 display a map of significant clusters overlaid on a standard-space brain, in an array of coronal and axial slices respectively. A similar cluster pattern was evident when images were aligned so that the right side of the brain was contralateral to the side of greater PD motor symptom severity in all subjects; although largely symmetrical, cluster size was slightly more extensive in the right-sided midbrain and left-sided temporal cortex (Supplementary Figs. 3 & 4).

### 1.3.2 Quantitative [^18^F]fallypride BP_ND_ differences in PD

**Fig. 3:**
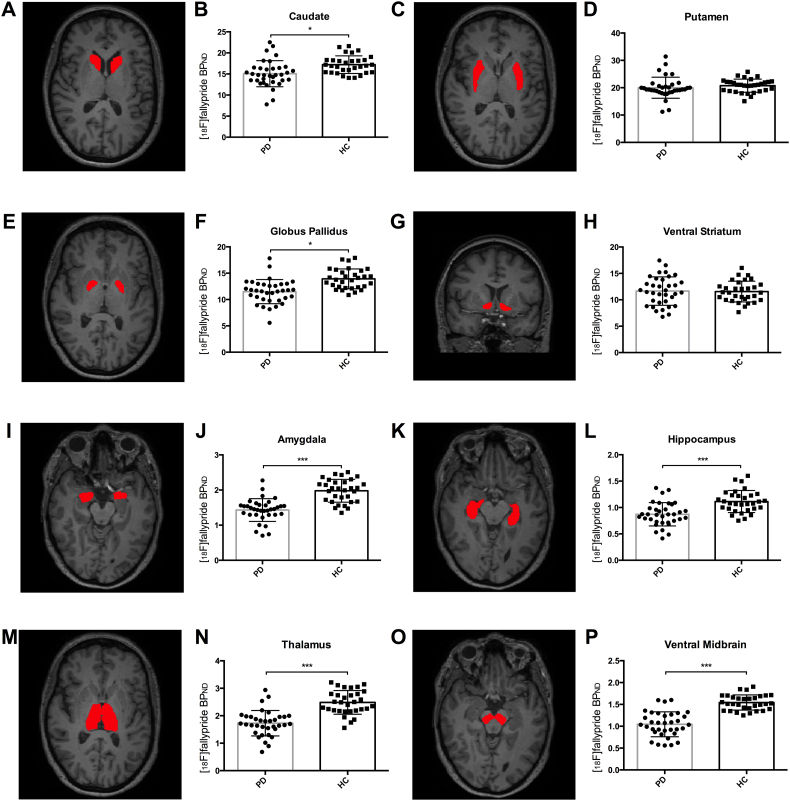
Mean regional [^18^F]fallypride binding potential analysis. (A–O) Representative coronal and axial slices for a single subject show an example of the manual segmentation routine for eight different subcortical structures, including (A) caudate, (C) putamen, (E) globus pallidus, (G) ventral striatum, (I) amygdala, (K) hippocampus, (M) thalamus, and (O) midbrain. (B–P) Bar graphs of the mean [^18^F]fallypride BP_ND_ in each corresponding region, with error bars representing the standard deviation of the mean, and scatterplots representing individual regional means. There were significant differences in mean regional BP_ND_ between the PD and HC group in the caudate (A–B), globus pallidus (E–F), amygdala (I–J), hippocampus (K–L), thalamus (M–N), and midbrain (O–P). No group differences were observed in the putamen (C–D) or ventral striatum (G–H).

To quantitate group differences in mean subcortical regional [^18^F]fallypride BP_ND_ we applied a GLM analytic approach, including group as an independent variable, and age, sex, and volume as covariates. In order of the magnitude of percent difference, significant BP_ND_ reductions in PD patients were evident in the ventral midbrain (*p* < 0.001; 39% decrease), amygdala (*p* < 0.001; 33% decrease), thalamus (*p* < 0.001; 30% decrease), hippocampus (*p* < 0.001; 22% decrease), globus pallidus (*p* = 0.002; 16% decrease), and caudate (*p* = 0.005; 11% decrease). No significant differences were evident in the putamen (*p* = 0.56; 2% difference), or ventral striatum (*p* = 0.66; 2% difference). (See Supplementary Table 3 for full description of mean regional binding potentials). Fig. 3 presents the individual and group mean regional BP_ND_ values for the ROIs, alongside segmentation visualization.

To quantify the extent of BP_ND_ differences in regions where voxel-wise distinctions were present, but that were not captured by the hand-drawn ROI analysis, we applied the AAL atlas to define the bilateral combined entorhinal and parahippocampal cortices, the inferior and middle temporal gyri, and the temporal pole. The locus coeruleus (LC) was defined by uniting six contiguous 2 mm boxes bilaterally, centered in standard space (MNI) according to the mean values on the *x* and *y* axis as discussed by Isaias et al. (2011) and Keren et al. (2009). This approach revealed PD-induced BP_ND_ decreases of a similar magnitude to those observed in other extrastriatal areas: LC (27% decrease), entorhinal and parahippocampal cortices (19% decrease), inferior (25% decrease) and middle (23% decrease) temporal gyri, and temporal pole (24% decrease). (See Supplementary Table 5 for full description of mean regional binding potentials, and Supplementary Figs. 1 and 2 for depiction of the segmentation protocol as well as group and individual regional means).

### 1.3.3 Relationships between fallypride binding potential and PD motor severity

The relationship of BP_ND_ to MDS-UPDRS Parts II and III-Off scores was assessed using a voxel-wise analysis while covarying for age. We observed a positive relationship between [^18^F]fallypride BP_ND_ and UPDRS Part III-Off in a right hemisphere cluster localized to the globus pallidus and anterior putamen, and a left hemisphere cluster localized to the posterior putamen. No significant clusters were observed when BP_ND_ was tested for association with UPDRS Part II scores. Supplemental Fig. 4 displays a map of significant clusters overlaid on a standard-space map. When images were aligned so that the right side of the brain was contralateral to the side of increased PD motor symptom severity in all subjects, a significant cluster was evident only in the left-sided putamen and globus pallidus (Supplementary Fig. 6).

### 1.3.4 Regional relationships between fallypride binding potential and PD severity

**Fig. 4:**
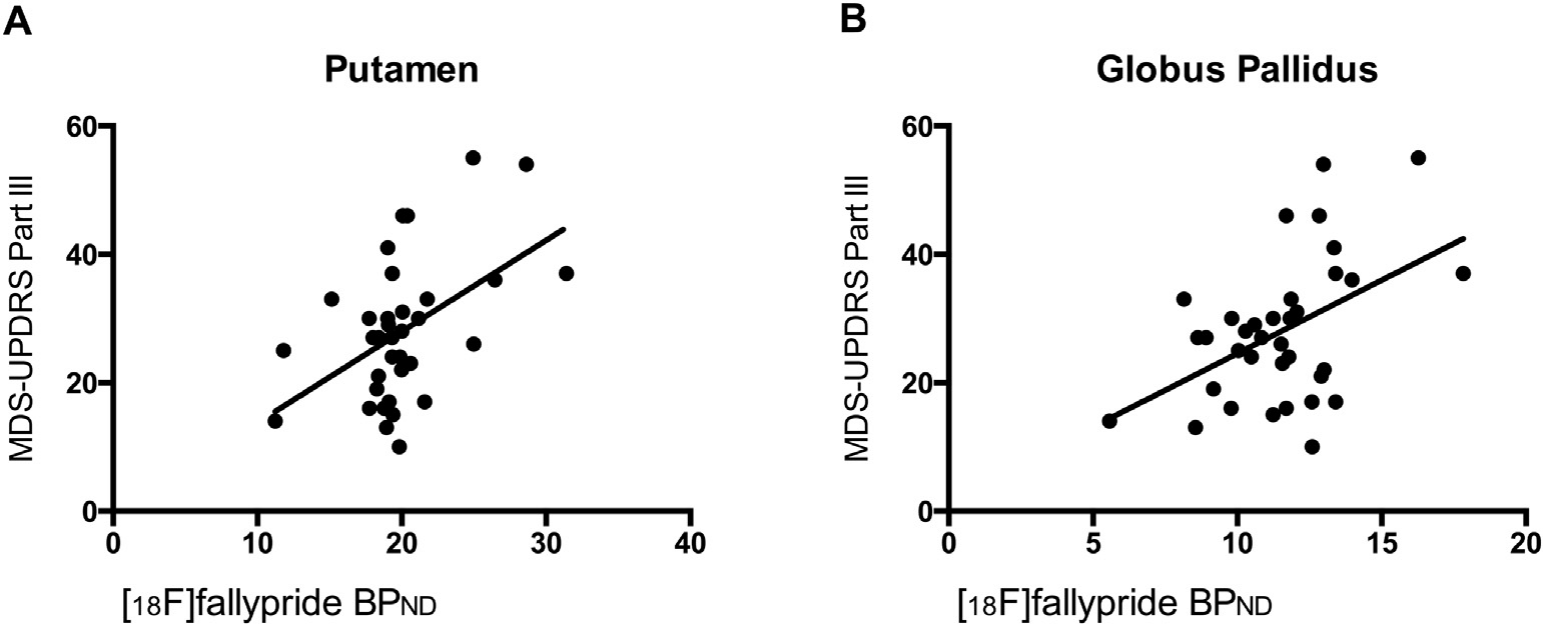
MDS-UPDRS Part III vs. [^18^F]-fallypride binding potential. Scatterplots of BP_ND_ (y-axis) vs. scores on the MDS-UPDRS Part III off-medication (x-axis) fit with a linear regression for the PD group. Age, sex, ROI volume, disease duration, and LEDD were included as covariates in this analysis. A significant positive correlation between BP_ND_ and MDS-UPDRS Part III score was observed for the (A) putamen and (B) globus pallidus. No significant correlations were observed between BP_ND_ and MDS-UPDRS Part II for any ROI. This indicates that there is a positive relationship between D_2/3_ expression in the putamen and globus pallidus, and severity of PD motor symptoms.

To further evaluate the association between D_2/3_ BP_ND_ and motor severity, we assessed the relationship between [^18^F]fallypride BP_ND_ and UPDRS Part III-Off scores in the hand-defined bilateral putamen and globus pallidus, while covarying for factors that may influence motor severity. This included age, sex, ROI volume, disease duration, and LEDD. We observed a significant positive correlation between BP_ND_ and scores on the MDS-UPDRS Part III in the putamen (*r* = 0.488, *p* = 0.006) and globus pallidus (*r* = 0.449, *p* = 0.013). No significant association was present between MDS-UPDRS Part III and BP_ND_ in any other ROI. Again, we did not see a significant relationship between [^18^F]fallypride BP_ND_ and MDS-UPDRS Part II. Fig. 4 displays the graphs of MDS-UPDRS Part III score with BP_ND_ in the putamen and globus pallidus.

## 1.4 Discussion

We observed widespread reductions in D_2/3_ receptor binding in PD patients, indicating changes to D_2/3_ receptor availability in regions that relate to both the motor and nonmotor aspects of PD. The sites in which D_2/3_ BP_ND_ decreases were observed include basal ganglia, limbic, thalamic, and cortical regions, as well as the LC. Our discussion focuses on these D_2/3_ BP_ND_ group differences in the basal ganglia (and relationships with the severity of off-motor symptoms in PD), norepinephrine-related extrastriatal areas, and limbic and thalamocortical regions. Broadly, the pattern of BP_ND_ reduction in the basal ganglia is of a lower magnitude, indicative of a complex relationship between presynaptic receptor loss and postsynaptic receptor upregulation, while extrastriatal areas show larger differences between the PD and HC groups.

### 1.4.1 Technical considerations

The interpretation of data presented in this report requires the consideration of several technical parameters relevant to determining the neurobiological origin of PD-related changes reflected in [^18^F]fallypride BP_ND_ values. D_2/3_ receptors are expressed on dopaminergic neurons, where they can operate presynaptically as autoreceptors (Khan et al., 1998; Mercuri et al., 1992), or as postsynaptic receptors present on striatal medium spiny neurons and cortical pyramidal cells (Albin et al., 1989; Gaspar et al., 1995). Thus, a decrease in [^18^F]fallypride BP_ND_ values could potentially convey a loss of autoreceptors secondary to degeneration of mesotelencephalic dopaminergic neurons, or a decrease in postsynaptic D_2/3_ expression. However, the latter possibility appears unlikely to be a primary source of decline in receptor availability, as nigrostriatal dopaminergic denervation elicits a compensatory upregulation of postsynaptic receptors early in the course of PD (Falardeau et al., 1988). Considering that our subjects were relatively early in the course of motor parkinsonism, it is likely that the present report of decreased [^18^F]fallypride BP_ND_ is a measure of presynaptic D_2/3_ autoreceptor density, influenced by ongoing degeneration of dopaminergic neurons. Finally, interpretation of [^18^F]fallypride BP_ND_ must also account for the combined influence of long-term receptor expression and the level of dopamine in the synaptic cleft which competes with [^18^F]fallypride binding leading to an underestimation of the long-term D_2/3_ expression level.

### 1.4.2 Nigrostriatal and pallidal D2/3 expression: dopaminergic denervation and motor severity

Reduced D_2/3_ levels were observed in the ventral midbrain, caudate nucleus, and globus pallidus of PD patients. The initial and greatest loss of midbrain dopaminergic neurons in PD occurs in the ventrolateral substantia nigra (Gibb and Lees, 1991). It is therefore likely that the ventral midbrain reduction in BP_ND_ reflects nigral dopaminergic cell loss (Gibb and Lees, 1991), and the consequent loss of somatodendritic D_2/3_ autoreceptors. In contrast, D_2/3_ BP_ND_ did not differ in the putamen and ventral striatum between PD and HC subjects. While the loss of presynaptic terminal autoreceptors due to dopaminergic cell death likely accounts for the decrease in BP_ND_ observed in several regions (caudate and globus pallidus), D_2/3_ BP_ND_ is unchanged in certain other areas (putamen and ventral striatum), although these regions also suffer dopaminergic denervation. This is particularly striking in the putamen where dopaminergic denervation is typically greatest. The lack of significant differences between subjects with PD and control subjects presumably reflects a combination of postsynaptic receptor upregulation (Falardeau et al., 1988), combined with a decrease in terminal autoreceptors and extracellular DA levels. Post-synaptically, long-term binding decrements over the course of PD likely involve the loss of D_2/3_ expressing dendritic spines (Stephens et al., 2005; Zaja-Milatovic et al., 2005) a concept further emphasized by past evidence of putaminal atrophy (Tanner et al., 2017). In our hands, the combined influence of these factors on D_2/3_ in the putamen sums to no significant change. This may explain equivalent putaminal BP_ND_ results between patients and controls, but divergent values in the caudate. Because ventral midbrain dopaminergic cell death occurs in the ventrolateral substantia nigra (containing cell bodies from which the dopaminergic projection to the more dorsolateral and posterior putamen emanates), the greatest DA reductions more heavily affect the putamen than the caudate; these losses likely exceed a critical threshold and produce notable receptor upregulation in the region (Gibb and Lees, 1991; Haber et al., 2000; Rinne et al., 1993; Rinne et al., 1995). By contrast, either this threshold is not reached in other brain regions, or those areas lack the same compensatory upregulation that occurs in the putamen.

In addition to differences in long-term receptor expression, the lack of an observed BP_ND_ difference in the putamen could also be affected by acute reductions in synaptic dopamine, which may contribute to the positive correlation between motor symptoms and D_2/3_ binding in the region, which is likely weighted on the former factor. Patients with more severe motor symptoms after standardized dopamine withdrawal presumably have less endogenous synaptic dopamine levels after medication washout, resulting in less competition with [^18^F]fallypride binding. This concept could be further emphasized by the finding that this positive association was present only on the left hemisphere of the brain, following alignment of all BP_ND_ images so that more severe motor symptoms were expressed in the right hemi-body. The fact that the correlation is present on the side of the dopaminergic system less affected by denervation, where DA levels are likely more dynamic and sensitive to the presence of medication, could strengthen the association between observed striatal BP_ND_ and synaptic DA. Future studies employing pharmacological methods of manipulating dopamine levels may clarify this relationship between endogenous dopamine, regional differences in D_2/3_ receptor binding, and motor severity.

Our findings of D_2/3_ reductions in the globus pallidus are similar to previous observations in a [^11^C]-(+)-PHNO study of PD reporting significantly reduced binding in comparison to control subjects (Boileau et al., 2009). However, this is the first study to indicate a relationship between motor symptoms and pallidal D_2/3_ expression after medication washout. This association is in agreement with a recent study linking external globus pallidus function to motor severity in dopaminergic denervation mouse models (Mastro et al., 2017).

### 1.4.3 D_2/3_ receptor expression in regions receiving dense noradrenergic innervation

We also found D_2/3_ BP_ND_ changes in a number of regions that are classically known as noradrenergic sites, and receive less dense dopaminergic inputs. In PD patients, D_2/3_ BP_ND_ was decreased in the LC, a structure containing a large population of noradrenergic neurons that provide most norepinephrine in the forebrain. This effect was also present in the hippocampus, as well as several cortical sites in which norepinephrine concentrations are greater than those of dopamine (entorhinal/parahippocampal cortices, and temporal pole). While nigrostriatal denervation bears a clear link with the motor symptoms of PD, the association between the progressive loss of the noradrenergic system and many non-motor features has been amply described (Cumming and Borghammer, 2012). The degeneration of noradrenergic LC neurons in PD has long been known (German et al., 1992) and more recently the presence of α-synuclein inclusions in the LC has been reported (Braak et al., 2001). Moreover, noradrenergic loss in several cortical areas, including the prefrontal cortex, is comparable to or greater than the corresponding decrease in dopamine content in these structures in MPTP-induced parkinsonism (Elsworth et al., 1990) and in idiopathic PD (Scatton et al., 1983). D_2_ receptors are expressed on LC neurons (Mansour et al., 1990), suggesting that the degeneration of LC neurons may underlie the decrease in BP_ND_ due to postsynaptic D_2/3_ loss in subjects with PD. However, dopamine also positively regulates LC noradrenergic activity via reciprocal projections between ventral midbrain dopaminergic neurons and the LC (Deutch et al., 1986; Guiard et al., 2008). As such, the loss of presynaptic D_2/3_ expressed on dopaminergic midbrain projections to the LC may also contribute to the decrease in D_2/3_ binding in the region.

In the hippocampus, the density of dopaminergic innervation from the midbrain is sparse (Otmakhova et al., 2013), meaning that decreased BP_ND_ there is less likely due to presynaptic D_2/3_. Recent evidence has indicated that noradrenergic projections to the hippocampus and associated cortical regions may account for a large proportion of dopamine release in these structures (Devoto et al., 2005; Smith and Greene, 2012). Combined with the finding that norepinephrine may act as a D_2/3_ receptor agonist (Sanchez-Soto et al., 2016), the D_2/3_ BP_ND_ reductions in the LC, hippocampus, and medial temporal cortex could instead indicate widespread dysfunction of the noradrenergic system and its terminal fields, a well-described. Degeneration of this system, centering on the LC, is suggested as a major contributor to cognitive and other behavioral non-motor symptoms of PD (Benarroch, 2017; Rommelfanger and Weinshenker, 2007; Weiss et al., 1986).

### 1.4.4 Limbic and thalamocortical D2/3 expression in PD

The dopaminergic innervation of the amygdala arises from ventral midbrain neurons largely targeting the basolateral complex (Garcia-Amado and Prensa, 2013), where D_2/3_ receptors regulate reward learning processes (Berglind et al., 2006; Di Ciano and Everitt, 2004). The amygdala is known to be vulnerable to PD pathology, given the presence of Lewy body inclusions and susceptibility to atrophy (Braak et al., 1994; Junque et al., 2005). This pattern of degeneration is linked to the manifestation of depression in PD, where symptom severity correlates with reduced amygdalar volume (van Mierlo et al., 2015), and apparent hypofunction (Diederich et al., 2016; Sheng et al., 2014). Deficits in emotional processing constitute another non-motor symptom commonly attributed to dopaminergic innervation of the amygdala (Bowers et al., 2006), supported by the apparent influence of DA medication state on both behavioral and amygdalar hemodynamic response to emotional stimuli (Tessitore et al., 2002). D_2/3_ agonist medications show efficacy in ameliorating depression in PD (Barone et al., 2006), an effect that could potentially be induced by targeting D_2/3_ in the amygdala, where D_2/3_ agonists significantly displace receptor-bound [^11^C]FLB-457 (Ishibashi et al., 2011). Therefore, altered D_2/3_ BP_ND_ in the amygdala may account for some of the non-motor symptoms related to PD-depression. The extent of D_2/3_ receptor reductions to this region in our cohort of non-depressed patients suggests that this is an early area of vulnerability in PD which may contribute to subsequent depression risk.

The thalamus expresses notable D_2/3_ populations in the midline intralaminar and mediodorsal nuclei (Garcia-Cabezas et al., 2007; Rieck et al., 2004). Due to the selection of an ROI in the middle of the superior-inferior thalamic axis, BP_ND_ in this ROI is heavily weighted towards D_2/3_ expression in the medial thalamus. Although the function of thalamic D_2/3_ receptors is largely unexplored, the role of altered thalamocortical networks in the manifestation of motor symptoms of PD is well established (Sarnthein and Jeanmonod, 2007). Anatomically, D_2/3_ populations in the thalamus integrate information from mesocortical and limbic regions and project to frontal cortical areas (Goldman-Rakic and Porrino, 1985). Previous [^11^C]FLB-457 PET studies that show reductions in thalamic D_2/3_ binding also restrict these regional reductions to advanced, but not early, PD populations (Kaasinen et al., 2003; Kaasinen et al., 2000; Ko et al., 2013). In contrast, however, the degeneration of neurons in the centromedian-parafascicular complex occurs in both Hoehn and Yahr stage I/II and stage III/IV (Henderson et al., 2000), suggesting that the loss of [^11^C]FLB-457 binding may preferentially reflect the loss of certain afferents to these intralaminar nuclei. In a similar manner to regions already described, losses of presynaptic autoreceptors could also play a role.

We do not observe differences in frontal cortical areas. Previous [^11^C]FLB-457 studies have indicated reductions that localize to these regions (Kaasinen et al., 2003; Kaasinen et al., 2000; Ko et al., 2013); however, previous preclinical experiments utilizing MPTP models have not always proved consistent with this evidence (Gnanalingham et al., 1993). Our result could be a product of the pharmacodynamic distinctions between [^11^C]FLB-457 and [^18^F]fallypride (Narendran et al., 2009). However, this also may be due to our cohort of moderately affected patients, in whom previously described frontal cortical D_2/3_ reductions are not manifest until later in the course of the illness. We did, however, uncover differences in D_2/3_ receptors in the perirhinal and parahippocampal areas. These regions are critically involved in memory and learning processes (Zola-Morgan et al., 1989), which are often impaired in PD patients (Nagano-Saito et al., 2005). Future investigations on the clinical relevance of these findings may improve the characterization of cognitive changes early in PD.

## 1.5 Conclusions

Although a decided advantage of our investigation was the number of patients with PD recruited to the study, our PD cohort included a larger proportion (~50%) of individuals with compulsive reward-based behaviors than would be expected in a typical PD population (Garcia-Ruiz et al., 2014). We noted differences between PD patients with and without compulsive behaviors only in the ventral striatum and putamen, which were not significantly different between the HC and overall PD group; the lack of significant differences in these areas were preserved even when the subgroups were compared with the HC group separately (see Supplementary Table 7). Thus, we believe this is not a significant limitation to the interpretation of changes in PD. Also, the generalizability of the sample is affected by the relatively specific subject selection criteria, including patients with mild to moderate PD, who could tolerate an extensive dopamine washout, and were without depression, dementia, or psychosis.

In light of the close proximity of basal ganglia structures, there is potential for partial volume errors. We especially considered this given the significant association between D_2/3_ BP_ND_ and UPDRS Part III in the putamen and globus pallidus, two adjacent structures. However, the presence of a mean D_2/3_ BP_ND_ difference between groups in the globus pallidus but not in the putamen, in addition to the broad localization of the cluster observed in the voxel-wise correlation analysis, gives us confidence that our results in both regions are largely distinct. Additionally, all native subject space analyses included ROI volume as a covariate, which likely decreases the confounding effect of divergent patterns of atrophy in adjacent areas.

As a cross-sectional study, the present work can only tentatively point to possible changes in D_2/3_ BP_ND_ changes that occur over the course of disease progression. Additionally, the use of D_2/3_ agonists by all those enrolled makes it difficult to disentangle the effects of chronic long-term medication exposure from those purely induced by PD. Although past studies have assessed changes to extrastriatal D_2/3_ binding and dopaminergic biomarkers over time (Kaasinen et al., 2003; Nandhagopal et al., 2009), future studies should longitudinally assess extrastriatal D_2/3_ expression in a de novo PD population. Relating non-motor symptoms (e.g. mood and cognitive impairment), to receptor-level changes in these regions will be necessary to define a causal relationship to non-motor symptoms in PD. Overall, our findings emphasize that PD is associated with widespread changes in the expression of D_2/3_ receptors, and point to increased scrutiny of the contribution of noradrenergic alterations as contributing to changes in dopaminergic systems in PD. These changes appear to affect regions implicated in the development of both motor and nonmotor features of the disease, where D_2/3_ losses proceed differently in the basal ganglia and extrastriatal areas.

## Funding and disclosure

This was supported by the National Institutes of Health/National Institute of Neurological Disorders and Stroke (R01NS097783, K23NS080988) and National Institute of Aging (R01AG044838); and CTSA award No. UL1TR000445 from the National Center for Advancing Translational Sciences. The authors declare no conflict of interest.

## Appendix A Supplementary data

The supplementary data section provides 1.) PET acquisition parameters; 2.) a full description of mean ROI volume and BPnd values; 3.) a measure of effect size for all ROI-based analyses; 4.) standard-space ROI visualization and standard-space ROI BPnd scatterplots; 5.) visualization of supplementary whole-brain voxel-wise analysis considering PD symptom laterality; 6.) visualization of supplementary whole-brain voxel-wise analysis evaluating correlation between BPnd and UPDRS Part III. Image 1
