# Supplementary material for: [18F]fallypride characterization of striatal and extrastriatal D2/3 receptors in Parkinson's disease
Source: Neuroimage Clin. 2018 Feb 10;18:433–42. doi: 10.1016/j.nicl.2018.02.010 (PMC5849871; doi:10.1016/j.nicl.2018.02.010)
Supplement: Supplementary file 1 — The supplementary data section provides 1.) PET acquisition parameters; 2.) a full description of mean ROI volume and BPnd values; 3.) a measure of effect size for all ROI-based analyses; 4.) standard-space ROI visualization and standard-space ROI BPnd scatterplots; 5.) visualization of supplementary whole-brain voxel-wise analysis considering PD symptom laterality; 6.) visualization of supplementary whole-brain voxel-wise analysis evaluating correlation between BPnd and UPDRS Part III. [file mmc1.docx]

**Supplementary Methods**


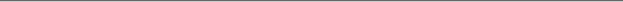


**Supplementary Table 1. PET acquisition time parameters.** The protocol for the second and third dynamic runs varied slightly between the PD and OHC cohort.

| **PET Frame** |
| --- |
| **Acquisition Times (secs)** |
| **Discovery STE Scan Protocol** |
|  |
| **Dynamic 1 (DY1)** |
| 0-15 |
| 15-30 |
| 30-45 |
| 45-60 |
| 60-75 |
| 75-90 |
| 90-105 |
| 105-120 |
| 120-150 |
| 150-180 |
| 180-210 |
| 210-240 |
| 240-270 |
| 270-300 |
| 300-360 |
| 360-420 |
| 420-480 |
| 480-540 |
| 540-600 |
| 600-660 |
| 660-810 |
| 810-1100 |
| 1110-1410 |
| 1410-1710 |
| 1710-2310 |
| 2310-2910 |
| 2910-3510 |
| 3510-4110 |
| BREAK |

| **PET Frame** | |
| --- | --- |
| **Acquisition Times (secs)** | |
| **Discovery STE Scan Protocol** | |
|  |  |
| **PD** | **OHC** |
| **Dynamic 2 (DY2)** | |
| 1500 sec length | 750 sec length |
| 1500 sec length | 750 sec length |
| - | 750 sec length |
| - | 750 sec length |
| BREAK | |
| **PD** | **OHC** |
| **Dynamic 3 (DY3)** | |
| 1800 sec length | 1200 sec length |
| 1800 sec length | 1200 sec length |
| - | 1200 sec length |

**Acquisition Protocol:**

***PD***

Break 1: 1005±187 sec, Break 2: 962±221 sec

Mean Start Times for DY2: 5115±187 sec; DY3: 9062±364 sec

***Healthy Controls***

Break 1: 1114±204 sec, Break 2: 1031±124 sec

Mean Start Times for DY2: 5224±204 sec; DY3: 9256±249 sec

**Supplementary Results**


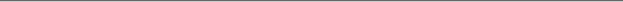


| ROI | Volume (PD) | Volume (HC) | p |
| --- | --- | --- | --- |
| Caudate | 1119±263 mm^3^ | 1132±154 mm^3^ | 0.47 |
| Putamen | 1697±362 mm^3^ | 1727±182 mm^3^ | 0.73 |
| Globus Pallidus | 598±94 mm^3^ | 464±90 mm^3^ | <0.0001 |
| Ventral Striatum | 456±144 mm^3^ | 465±106 mm^3^ | 0.33 |
| Amygdala | 764±153 mm^3^ | 618±126 mm^3^ | <0.0001 |
| Thalamus | 2010±516 mm^3^ | 1715±281 mm^3^ | 0.013 |
| Ventral Midbrain | 693±152 mm^3^ | 816±118 mm^3^ | 0.001 |
| Hippocampus | 3001±345 mm^3^ | 3062±317 mm^3^ | 0.22 |

| ROI | BP_ND_ (PD)* | BP_ND_ (HC)* | BP_ND_ (PD) | BP_ND_ (HC) | p |
| --- | --- | --- | --- | --- | --- |
| Caudate | 15.072±0.523 | 17.211±0.377 | 15.267±0.405 | 16.990±0.431 | 0.005 |
| Putamen | 19.999±0.649 | 20.760±0.431 | 20.154±0.500 | 20.585±0.532 | 0.561 |
| Globus Pallidus | 11.498±0.387 | 13.929±0.339 | 11.681±0.380 | 13.722±0.410 | 0.002 |
| Ventral Striatum | 11.651±0.456 | 11.555±0.354 | 11.729±0.405 | 11.467±0.431 | 0.661 |
| Amygdala | 1.430±0.055 | 1.979±0.058 | 1.425±0.055 | 1.984±0.059 | <0.001 |
| Thalamus | 1.728±0.078 | 2.486±0.078 | 1.786±0.071 | 2.420±0.076 | <0.001 |
| Ventral Midbrain | 1.044±0.048 | 1.543±0.031 | 1.039±0.044 | 1.549±0.047 | <0.001 |
| Hippocampus | 0.871±0.037 | 1.113±0.038 | 0.882±0.035 | 1.101±0.037 | <0.001 |

**Supplementary Table 2. Mean ROI volumes.** Mean volumes are listed alongside standard deviations, in mm^3^. Several significant differences were found in structure size between the PD and HC groups, indicating that structural volume could be a confounding factor. As a result, ROI volume was included as a covariate in all subject-space analyses

**Supplementary Table 3. Mean ROI BP_ND_.** Uncorrected mean BP_ND_ is listed for each group alongside standard error of the mean, in the columns denoted by the asterisk. In the unmarked columns, age, volume, and sex corrected mean BP_ND_ is listed for each group alongside standard error of the mean. Significant differences were found in between the PD and HC groups in the caudate, globus pallidus, amygdala, thalamus, ventral midbrain, and hippocampus.

| ROI | η^2^ * | Unstandardized B |
| --- | --- | --- |
| Caudate | 0.183 | 1.723 |
| Putamen | 0.045 | 0.430 |
| Globus Pallidus | 0.286 | 0.622 |
| Ventral Striatum | 0.001 | -0.263 |
| Amygdala | 0.451 | 0.559 |
| Thalamus | 0.453 | 0.634 |
| Ventral Midbrain | 0.562 | 0.510 |
| Hippocampus | 0.256 | 0.220 |

**Supplementary Table 4. Group difference effect size.** η^2^ is listed for each ROI, where the asterisk denotes that these values correspond with the effect size of a Mann-Whitney U test that evaluated no covariates, preventing its inclusion in the “Results” section. Unstandardized B is also included, reflecting the effect size of a GLM including age, sex, and ROI volume as covariates.

**Supplementary Table 5. Mean ROI BP_ND_.** Uncorrected mean BP_ND_ is listed for each group alongside standard error of the mean, in the columns denoted by the asterisk. In the unmarked columns, age and sex corrected mean BP_ND_ is listed for each group alongside standard error of the mean. No significant differences were found in between the PD and HC groups in the putamen and ventral striatum, even when divided into ICB+ and ICB- subgroups

| ROI | BP_ND_ (PD)* | BP_ND_ (PD)* | BP_ND_ (PD) | BP_ND_ (HC) |
| --- | --- | --- | --- | --- |
| Locus Coeruleus | 0.522±0.034 | 0.522±0.034 | 0.535±0.027 | 0.701±0.029 |
| Entorhinal/Parahippocampal Cortices | 0.823±0.031 | 0.823±0.031 | 0.833±0.030 | 1.012±0.032 |
| Inferior Temporal Cortex | 0.510±0.023 | 0.510±0.023 | 0.512±0.032 | 0.658±0.034 |
| Middle Temporal Cortex | 0.380±0.022 | 0.380±0.022 | 0.384±0.028 | 0.482±0.030 |
| Temporal Pole | 0.505±0.030 | 0.505±0.030 | 0.522±0.028 | 0.663±0.030 |

| ROI | η^2^ * |
| --- | --- |
| Locus Coeruleus | 0.264 |
| Entorhinal/Parahippocampal Cortices | 0.235 |
| Inferior Temporal Cortex | 0.136 |
| Middle Temporal Cortex | 0.086 |
| Temporal Pole | 0.17 |

**Supplementary Table 6. Group difference effect size.** η^2^ is listed for each ROI, where the asterisk denotes that these values correspond with the effect size of a Mann-Whitney U test that evaluated no covariates, preventing its inclusion in the “Results” section.

| ROI | BP_ND_ (PD ICB+) | BP_ND_ (PD ICB-) | BP_ND_ (HC) | p |
| --- | --- | --- | --- | --- |
| Putamen | 19.017±0.704 | - | 20.155±0.519 | 0.204 |
| Putamen | - | 20.924±0.608 | 20.335±0.456 | 0.455 |
| Ventral Striatum | 10.838±0.618 | - | 11.447±0.456 | 0.434 |
| Ventral Striatum | - | 12.507±0.554 | 11.428±0.420 | 0.132 |

**Supplementary Table 7. Mean ROI BP_ND_.** Age and sex corrected mean BP_ND_ is listed for subjects with and without impulsive-compulsive behaviors, alongside standard error of the mean. Regions include the locus coeruleus, entorhinal and parahippocampal cortices, inferior and middle temporal gyri, and temporal pole.


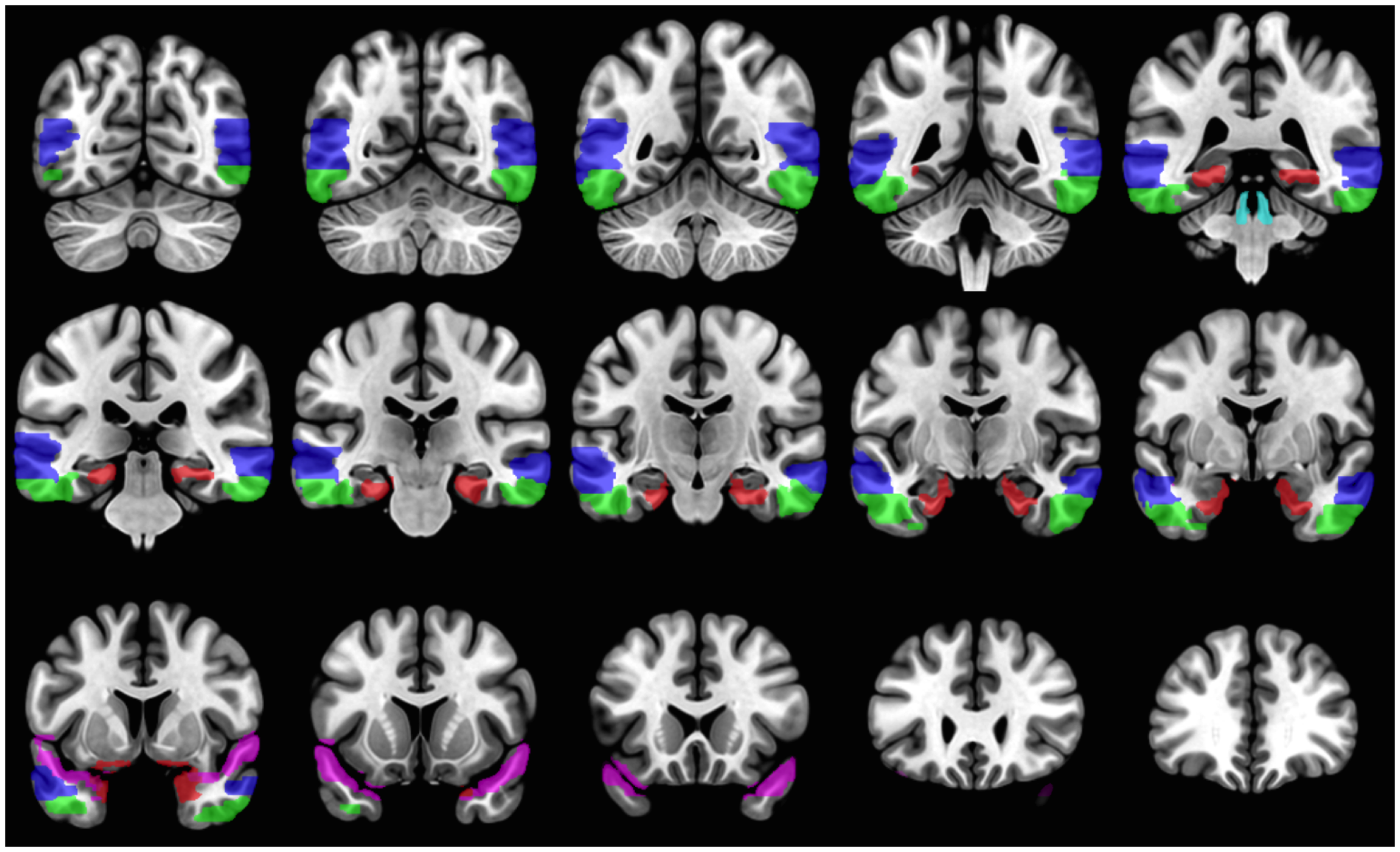


**Supplementary Figure 1. Post-hoc ROIs defined in standard space.** Map of ROIs used to quantify the size of [^18^F]-fallypride BP_ND_ differences in areas indicated as divergent between PD and HC subjects in the voxel-wise analysis, but not captured by the hand-drawn ROI analysis. The locus coeruleus appears cyan, the entorhinal and parahippocampal cortices appears red, the inferior and middle temporal cortices appear as green and blue respectively, and the temporal pole appears as violet. ROIs were defined using coordinate-based methods for the locus coeruleus (as highlighted in the Methods section), and using the Automatic Anatomical Labeling (AAL) for the cortex.


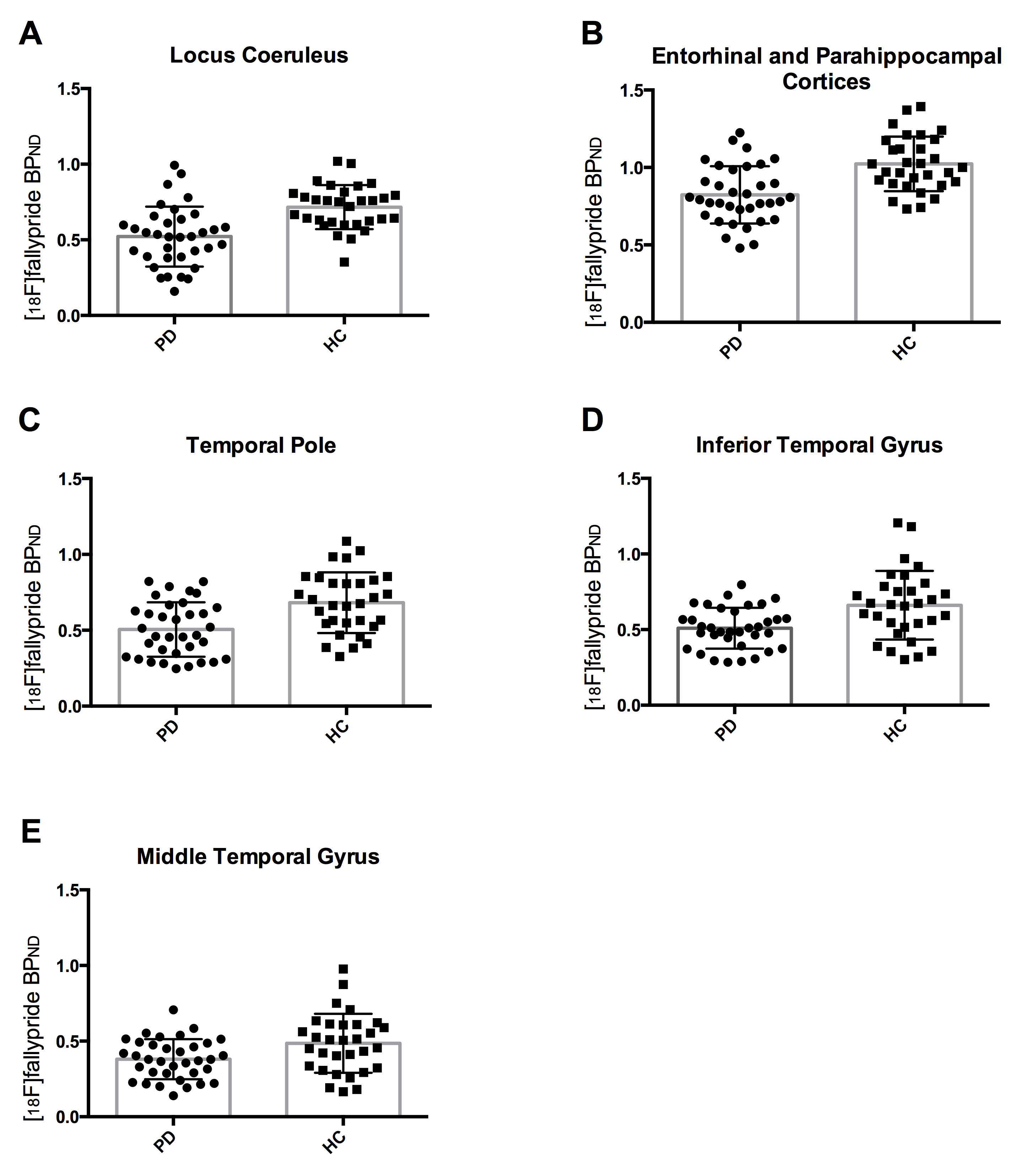


**Supplementary Figure 2. Mean regional [^18^F]fallypride binding potential for post-hoc ROIs.** Bar graphs of the mean [^18^F]fallypride BP_ND_ in each corresponding region, with error bars representing the standard deviation of the mean, and scatterplots representing individual regional means. These areas were selected in order to quantify regional differences between PD and HC subjects in areas identified as important in the voxel-wise analysis. ROIs were defined using coordinate-based methods for the locus coeruleus (as highlighted in the Methods section), and using the Automatic Anatomical Labeling (AAL) for the cortex. Areas examined include (A) locus coeruleus, (B) entorhinal and parahippocampal cortices, (C) temporal pole, (D) inferior temporal gyrus, and (E) middle temporal gyrus. Percent differences are reported in the Results section.


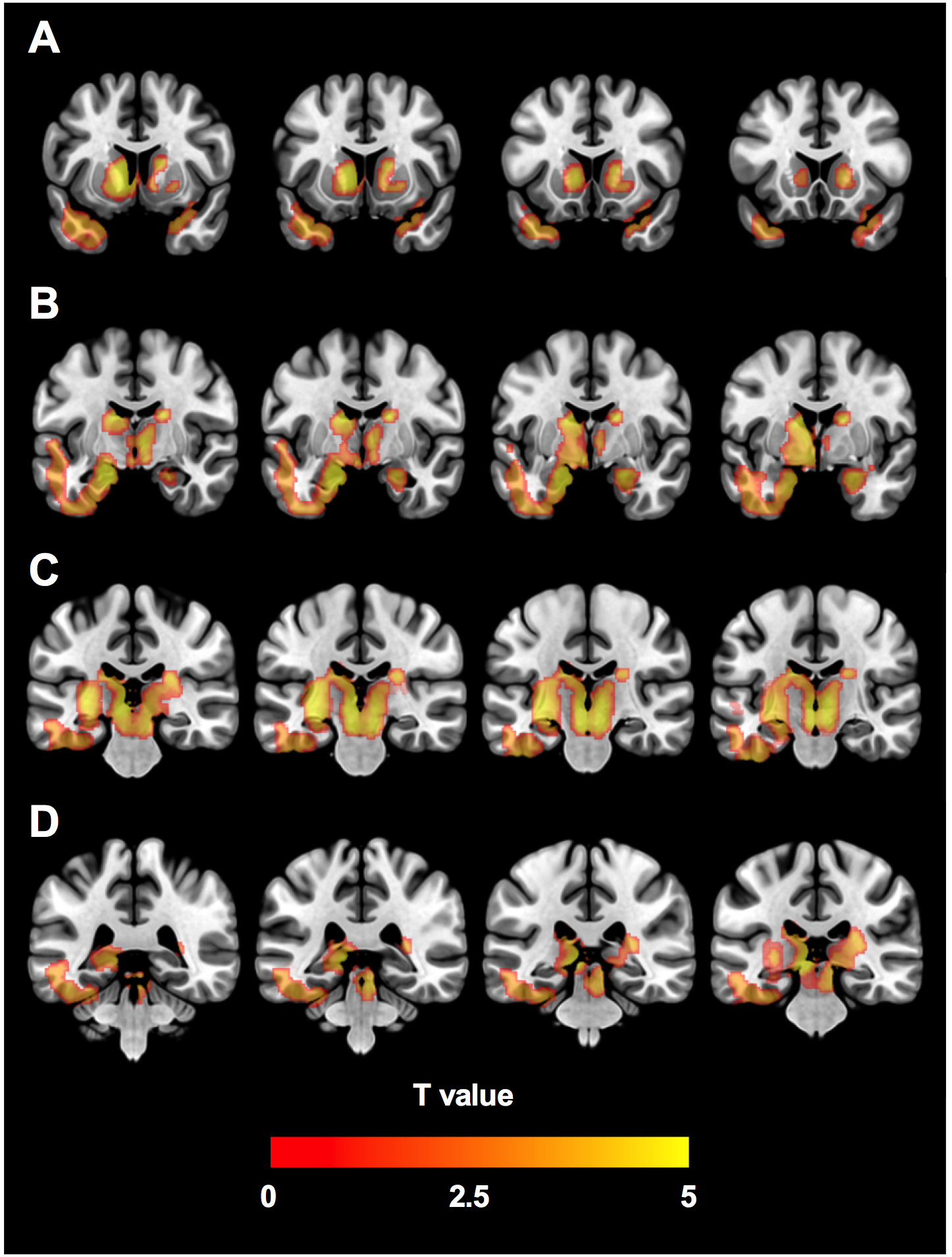


**Supplementary Figure 3. Voxel-wise [^18^F]fallypride binding potential analysis with laterality adjustment.** Map of significant clusters where [^18^F]-fallypride BPnd was reduced in PD, overlaid on coronal slices of an MNI template brain. BP_ND_ maps for subjects that exhibited right-side dominant PD symptoms were flipped across the y-axis of the axial plane. All survived cluster-level FDR correction at *P*<0.05, and localize to areas including (A) the striatum, globus pallidus, and temporal cortex, (B) the amygdala and hippocampus, (C) the ventral midbrain and thalamus, and (D) the locus coeruleus. Clusters appear to be more extensive on the left side of the brain in extrastriatal cortical and subcortical areas, and in the right-sided midbrain.


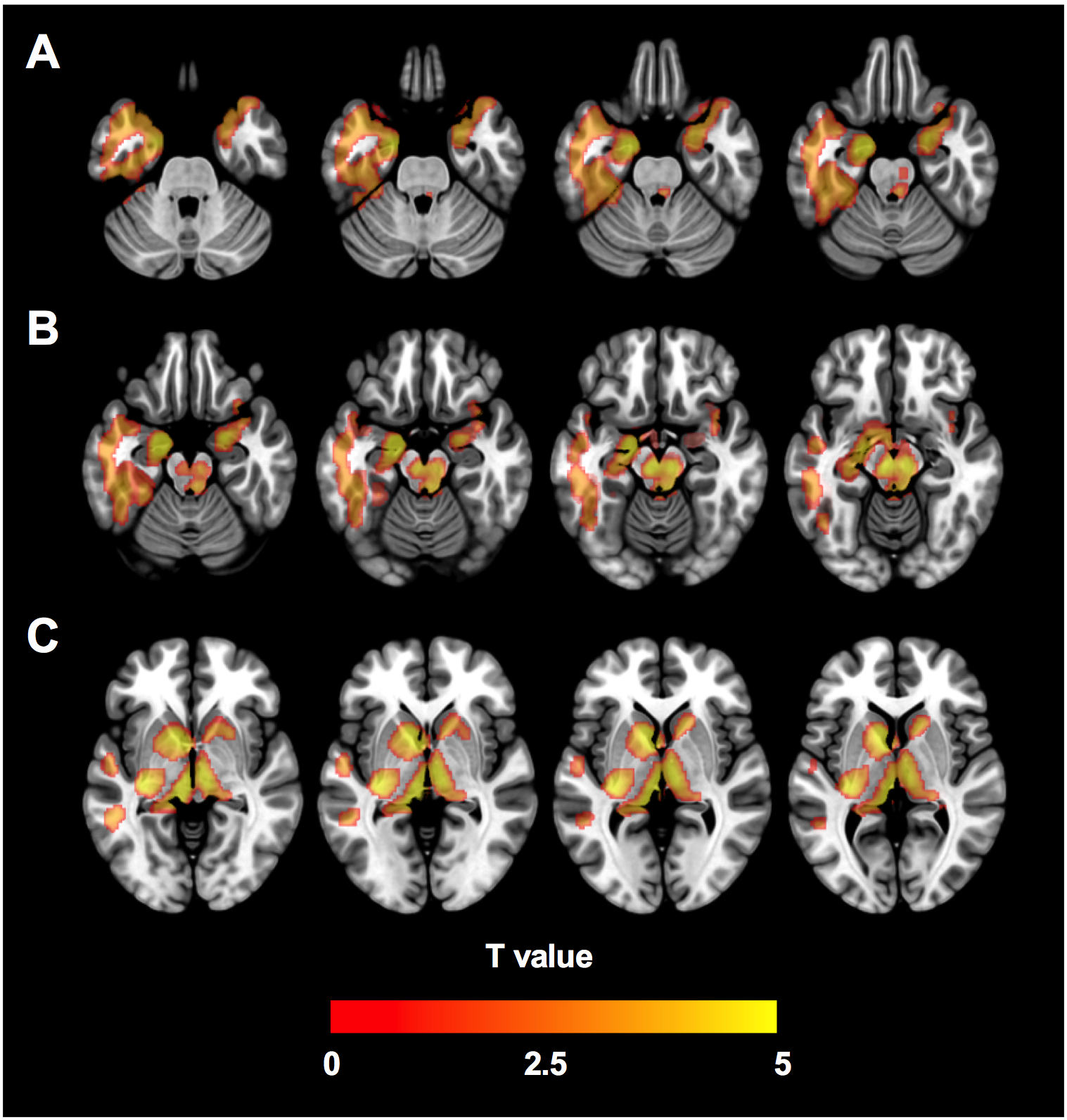


**Supplementary Figure 4. Voxel-wise [^18^F]fallypride binding potential analysis with laterality adjustment.** Map of significant clusters where [^18^F]-fallypride BPnd was reduced in PD, overlaid on axial slices of an MNI template brain. BP_ND_ maps for subjects that exhibited right-side dominant PD symptoms were flipped across the y-axis of the axial plane. All survived cluster-level FDR correction at *P*<0.05, and localize to areas including (A) the temporal cortex, (B) the amygdala, hippocampus, ventral midbrain, and locus coeruleus, and (C) the thalamus, striatum, and globus pallidus. Clusters appear to be more extensive on the left side of the brain in extrastriatal cortical and subcortical areas, and in the right-sided midbrain.


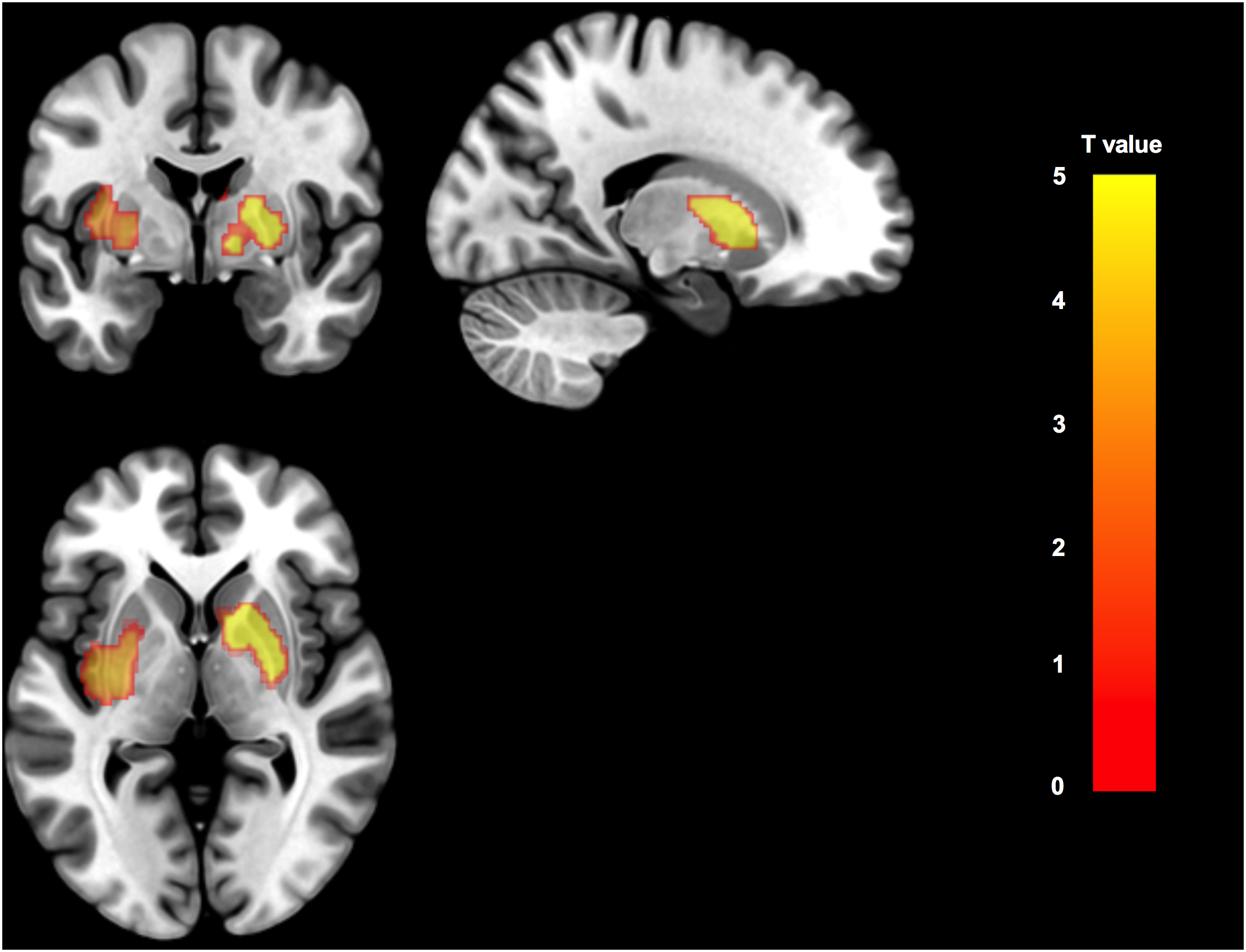


**Supplementary Figure 5. MDS-UPDRS Part III association with [^18^F]-fallypride binding potential.** Map of significant cluster where [^18^F]-fallypride BPnd correlated positively with MDS-UPDRS Part III while covarying for age, sex, disease duration, and LEDD, overlaid on coronial, axial, and sagittal slices of an MNI template brain. All survived cluster-level FDR correction at *P*<0.05, and localize to areas including the right-sided putamen and globus pallidus, and left-sided putamen.


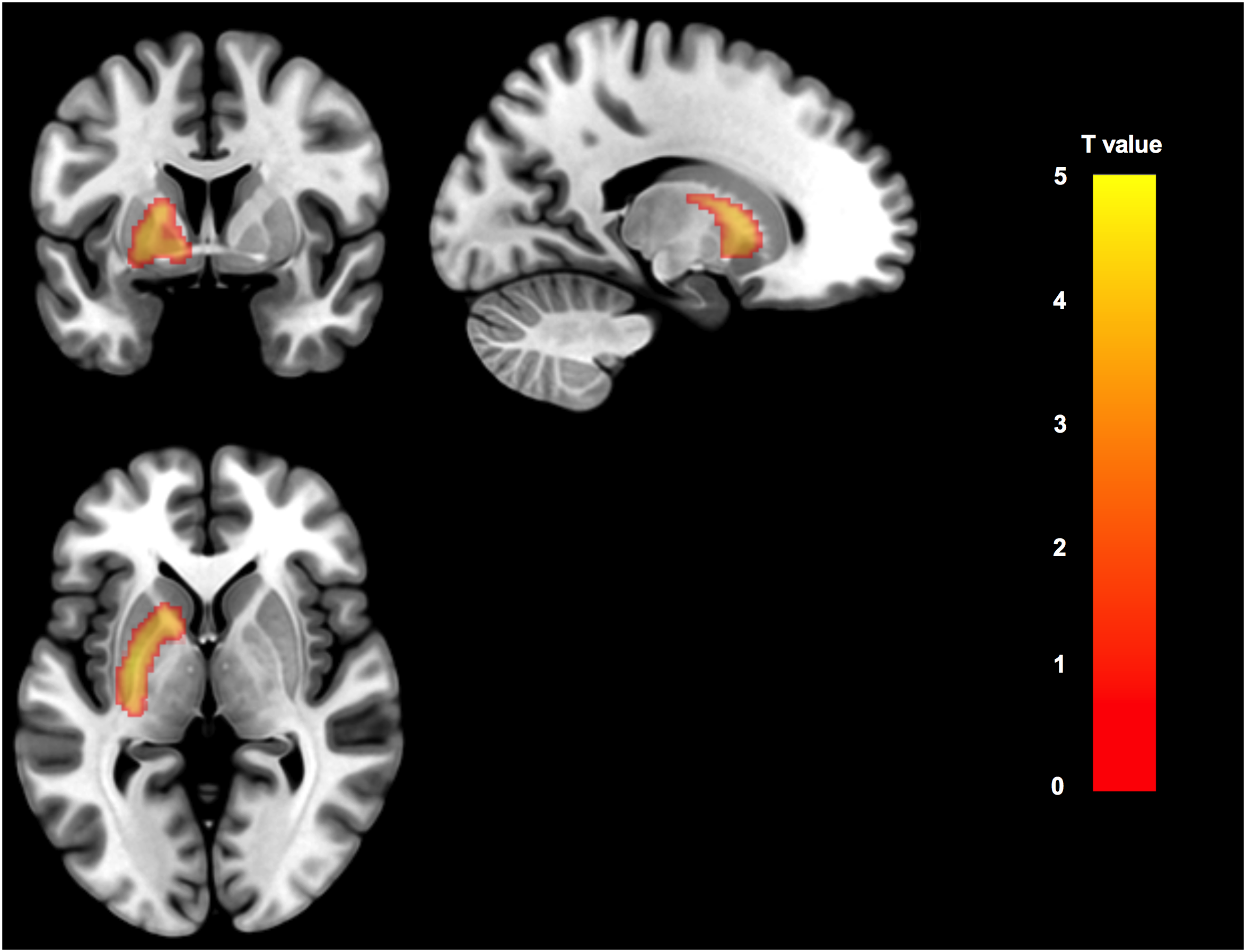


**Supplementary Figure 6. MDS-UPDRS Part III association with [^18^F]-fallypride binding potential with laterality adjustment.** Map of significant cluster where [^18^F]-fallypride BPnd correlated positively with MDS-UPDRS Part III while covarying for age, sex, disease duration, and LEDD, overlaid on coronial, axial, and sagittal slices of an MNI template brain. BP_ND_ maps for subjects that exhibited right-side dominant PD symptoms were flipped across the y-axis of the axial plane. All survived cluster-level FDR correction at *P*<0.05, and localize to areas including the right-sided putamen and globus pallidus, and left-sided putamen.
